# Securing recruitment and obtaining informed consent in minority ethnic groups in the UK

**DOI:** 10.1186/1472-6963-8-68

**Published:** 2008-03-30

**Authors:** Cathy E Lloyd, Mark RD Johnson, Shanaz Mughal, Jackie A Sturt, Gary S Collins, Tapash Roy, Rukhsana Bibi, Anthony H Barnett

**Affiliations:** 1 Faculty of Health & Social Care, The Open University, Milton Keynes, UK; 2 Mary Seacole Research Centre, De Montfort University, Leicester, UK; 3 Diabetes Centre, Birmingham Heartlands Hospital, Birmingham, UK; 4 Health Sciences Research Institute, Warwick Medical School, Coventry, UK; 5 Centre for Statistics in Medicine, University of Oxford, Oxford, UK; 6 Department of Medicine, University of Birmingham, Undergraduate Centre, Birmingham Heartlands Hospital, Bordesley Green East, Birmingham, B9 5SS, UK

## Abstract

**Background:**

Previous health research has often explicitly excluded individuals from minority ethnic backgrounds due to perceived cultural and communication difficulties, including studies where there might be language/literacy problems in obtaining informed consent. This study addressed these difficulties by developing audio-recorded methods of obtaining informed consent and recording data. This report outlines 1) our experiences with securing recruitment to a qualitative study investigating alternative methods of data collection, and 2) the development of a standardised process for obtaining informed consent from individuals from minority ethnic backgrounds whose main language does not have an agreed written form.

**Methods:**

Two researchers from South Asian backgrounds recruited adults with Type 2 diabetes whose main language was spoken and not written, to attend a series of focus groups. A screening tool was used at recruitment in order to assess literacy skills in potential participants. Informed consent was obtained using audio-recordings of the patient information and recording patients' verbal consent. Participants' perceptions of this method of obtaining consent were recorded.

**Results:**

Recruitment rates were improved by using telephone compared to face-to-face methods. The screening tool was found to be acceptable by all potential participants. Audio-recorded methods of obtaining informed consent were easy to implement and accepted by all participants. Attrition rates differed according to ethnic group. Snowballing techniques only partly improved participation rates.

**Conclusion:**

Audio-recorded methods of obtaining informed consent are an acceptable alternative to written consent in study populations where literacy skills are variable. Further exploration of issues relating to attrition is required, and a range of methods may be necessary in order to maximise response and participation rates.

## Background

The health status of minority ethnic groups in the U.K. is often compromised [1-3]. This is starkly demonstrated in Type 2 diabetes, where the prevalence is at least 5 times greater in South Asians living in the U.K. compared to white Europeans [4]. Type 2 diabetes is a chronic condition that requires self-management on a daily basis, along with support from a range of health care professionals. There are specific concerns with regard to supporting the appropriate self-management of the condition in minority ethnic groups, due to particular cultural and communication difficulties [3-8].

Research studies to improve both health service delivery and self-care within minority ethnic populations may be compromised because established ways of collecting data are often inappropriate for these groups [9,10]. High levels of illiteracy and lack of fluency in English are two key issues. Furthermore, in particular South Asian groups in the U.K. there is no agreed written form of the main spoken language; two such groups being those from the Sylhet region of Bangladesh, whose main language is Sylheti, and those from the Kashmir region of Pakistan whose main language is Mirpuri. Both these groups are well-represented (more than 50% of patients) in the diabetes out-patient department of Birmingham Heartlands Hospital and have been the focus of a recent intervention study in diabetes complications risk factor reduction [11]. In that study difficulties were encountered with the completion of standard forms of data collection, specifically traditional paper-and-pencil methods of completing questionnaires [12].

Obtaining informed consent is a requirement for all research studies within the NHS, and strict guidance on patient information sheets and consent forms is given by the National Research Ethics Service of the National Patient Safety Agency [13]. Previous research has suggested that recruiting and obtaining informed consent from individuals from minority ethnic groups can be challenging [14-16]. Patients' understanding of the importance of giving consent in any situation, for example prior to surgery, may be limited in some populations [17]. It follows that this may well be an issue in research and may be one explanation as to why many studies have low proportions of participants from minority ethnic backgrounds [18]. Indeed added responsibilities are often placed upon those researching in areas of high illiteracy and where understanding of the issues may be problematic, in terms of having to devise more innovative ways of carrying out studies [15], but also in terms of presenting consistent patient information prior to the obtaining of consent. However, as Bhutta [14] states, the notion of illiteracy does not mean that potential participants are unable to comprehend complex information, but it does mean that information may need to be presented in alternative ways. In all the international guidelines on informed consent, including those of the World Medical Association and the European Union guidelines, written consent is considered preferable, with verbal consent appropriate only where participants are non-literate [14]. To date it is our impression, based on a thorough search of the literature, that there appears to be a dearth of published studies where alternative methods of delivering patient information and obtaining informed consent are reported, and their effectiveness remains to be assessed. As Boulton and Parker note [19] whilst there has been increasing concern about informed consent in recent years, the focus of this has largely been on biomedical research, and in particular in relation to access and use of medical records. Where attention has turned to research outside biomedicine such as in the social sciences, current discussion concerns the nature of informed consent, for example in qualitative research where it might not be possible to predict how the research will develop, rather than on the methods through which informed consent might be obtained.

Successful involvement of minority ethnic groups in research can depend on the type of data collection planned. In a study of South Asians with diabetes (the UK Asian Diabetes – UKAD – Study [11]) the collection of questionnaire data pertaining to diabetes self-management was found to be problematic, although recruitment of individuals to the study was straightforward. Clinical data was easily collected, but the collection of psychosocial data was compromised as a high proportion of those approached were unable to complete standard questionnaires without the assistance of support workers [12]. Following these experiences, a study was designed to develop new ways of collecting data in South Asians with diabetes. An integral part of the study was to develop standard ways of obtaining informed consent from people from minority ethnic backgrounds who were non-literate, which could be applied to other minority ethnic groups involved in research. The development of a standardised process of obtaining consent in situations such as these could lead to greater involvement of non-literate people in research in general. This report outlines the results of our recruitment process and methods of obtaining informed consent in two groups of South Asians: Sylheti and Mirpuri speakers, whose main language does not have an agreed written form. The design of this exploratory study was based upon our previous experiences with the UKAD study, drawing on the local knowledge and expertise of those involved in collecting data for that project [20].

## Methods

After receiving ethical approval from the local Research Ethics Committee, adults with Type 2 diabetes were recruited, either face-to-face or by telephone, via the Birmingham Heartlands Hospital diabetes centre. Individuals who spoke either Sylheti or Mirpuri were invited to participate in five focus group meetings, which would take place over a four-month period, to consider the content and mode of delivery of two diabetes self-management questionnaires. The focus group sessions were gender and language specific, and were facilitated by one of 2 researchers along with either a lay member of the community (Sylheti groups) or an Asian link worker (Mirpuri groups). Sessions took place on weekdays, lasted approximately 90 minutes and participants received refreshments and their travel expenses. Focus group sessions 1 and 2 took place on the same day before and after lunch, and included discussion on the content and format of the two questionnaires including the language, concepts and terms used. Items on the questionnaires were read out by the researchers and discussed in both the written and non-written languages so that both literate and non-literate individuals could participate fully. Focus group session 3 took place in the morning approximately three months later and consisted of a preliminary testing of the adapted questionnaires (based on the findings of the first 2 focus group sessions) in both written and audio format. Focus group sessions 4 and 5 took place on the same day one month later, with a break for lunch in between, and consisted of the testing out of four alternative formats for completing questionnaires. The alternative formats were (i) pen & paper self-completion, (ii) pen & paper assisted completion in a spoken language, (iii) partially-assisted completion in a spoken language, and (iv) independent audio-delivery in a spoken language. Only literate individuals were able to test the first format, and all participants tested methods ii, iii and iv.

Initially patients attending the diabetes clinic who spoke Sylheti or Mirpuri were identified by two researchers (one male Sylheti speaker, one female Mirpuri speaker) who were fluent in both English and either Sylheti or Mirpuri in consultation with the Diabetes Centre Asian Link Workers. The only exclusion criterion was a diagnosis of diabetes of less than one year, so as to ensure that diabetes education had taken place, and that individuals had had time to come to terms with their diagnosis. Following difficulties in face-to-face recruitment, adults with Type 2 diabetes who were already participating in the UKAD study [11] were approached via the telephone. The aim was to recruit a maximum of 10 individuals to each group so that each focus group could be gender-specific, with no more than half of each group being literate in order to ensure that both written and non-written versions of the questionnaires could be considered simultaneously. A sensitive assessment of literacy skills in both English and Bengali/Urdu was made by the researchers during recruitment (see Table 1). This was developed by the researchers in consultation with the Asian link workers, based on their knowledge of the study populations involved.

**Table 1:** Screening questions pertaining to ethnicity and language/literacy.

What is/was your country of origin/ethnic origin?	□ Bangladesh □ Pakistan □ Others (non-eligible) .........
Which part of Bangladesh/Pakistan are/were you originally from?	□ Sylhet □ Dhaka □ Punjab □ Mirpur □ Other...................
Can you speak in English? Can you read and write in English ?	□ Yes □ No □ Yes □ No
Can you speak in Bengali/Urdu? Can you read and write in Bengali/Urdu?	□ Yes □ No □ Yes □ No
What language/dialect do you usually use at home?	□ Bengali □ Sylheti □ Urdu □ Mirpuri □ Others..................
What language/dialect do you usually use to speak with other people (friends, other members of same community/different community)	□ Bengali □ Sylheti □ Urdu □ Mirpuri □ Other .................

The screening questionnaire was developed to ensure sensitivity when asking about literacy skills. The literacy assessment tool was tested during the 2 pilot focus group meetings (consisting of 5 Sylheti men and 4 Mirpuri women) prior to initial implementation at face-to-face recruitment. Further consideration was given to the tool after the first week's recruitment, and an additional question was included in order to ascertain individual plans for holidays in the coming months, as this would impact on participation and there were times during the year when people were more likely to visit their country of origin. No further difficulties were encountered in the use of the screening tool and so no further adaptations were required.

Informed consent was obtained using audio-recorded patient information and participant responses. The patient information sheet was audio-recorded by the researchers on to audio-cassette in English, Sylheti and Mirpuri, and written versions were developed in Bengali and Urdu. Back-translations were obtained from two sources; an academic (Masters level education or higher) and a lay person (no known academic qualifications) known to the researchers. The final version of the information was biased towards the lay translation as it was felt to be more easily understood and would be more acceptable to our research participants. Details of the procedure for obtaining audio-recorded informed consent are given in Table 2.

**Table 2:** Procedure for obtaining audio-recorded informed consent.

1. Individuals were given the audio-recorded patient information to take home, or were sent the audio-tape if recruited via telephone. They were also given a written copy of the patient information sheet and consent form so that, if they wished, they or other family members could read the information. The written information was available in English, Bengali and Urdu. They were subsequently telephoned in order to establish if they had any concerns or queries.
2. Individuals were invited to attend the first focus group meeting 20 minutes early so that audio-recorded informed consent could be obtained on an individual basis.
3. Researchers enquired whether the audio-recorded patient information had been listened to and answered any questions or concerns. If necessary a further opportunity to listen to the audio-recording was provided prior to taking informed consent.
4. Researchers explained how informed consent was going to be taken as an audio-recording.
5. Individuals were asked to repeat the words on the consent form (read out by the researcher point by point), and asked if they understood and agreed with each point of the consent form.
6. Individuals were asked to state their name, the date and the time of giving their consent. A copy of the audio-recorded consent was given to the participant to take away with them.

Reactions to the obtaining of informed consent were noted down by the researchers. Prior to each focus group session participants were contacted by the researchers in order to confirm their attendance. Those who were unable to attend were asked if there was a particular reason for this, and if they would consider attending subsequent sessions. After the focus group sessions had been completed all participants were invited back to an 'end-of-project lunch', during which participants were thanked for their involvement in our research and informal feedback was obtained on potential ways to improve recruitment and attrition rates.

## Results

### Recruitment

**Figure 1:**
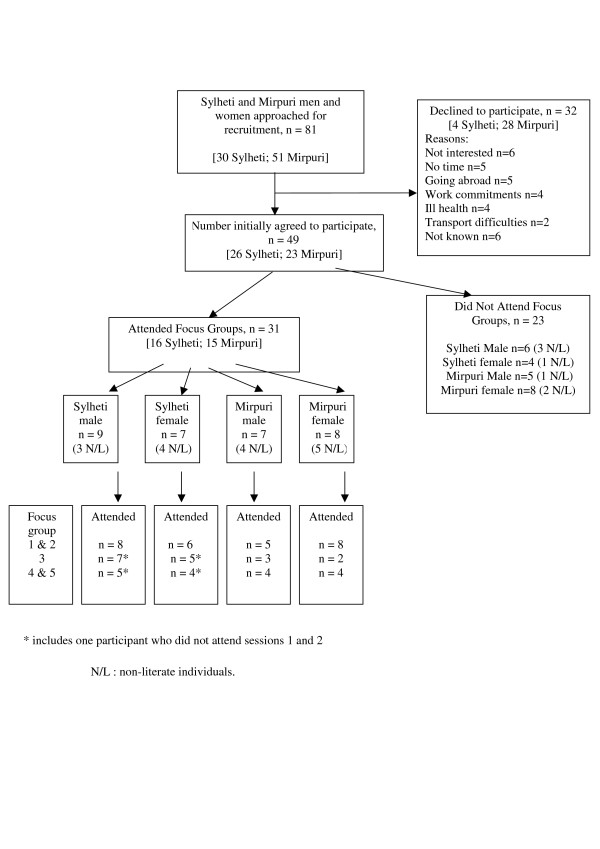
Flow of participants through the study.

After 7 weeks our aim of recruiting at least 10 people to each language/gender group was achieved, facilitated by the successful collaboration between the two researchers and the Asian Link Workers. A total of 30 Sylheti and 51 Mirpuri men and women were approached, with 87% (26/30) Sylheti and 45% (23/51) Mirpuri individuals agreeing to participate. Face-to-face recruitment at the diabetes clinic was problematic, with few people being eligible and those who were, unwilling to take time out during their clinic appointment to consider participating. Few Sylheti speakers attended the diabetes clinic. Although there were initial concerns with regards to recruiting people of the opposite gender or who were non-literate, this was not born out by the recruitment rates (Figure 1).

One researcher reported that her difficulties recruiting face-to-face may have been because of her young age and style of dress. She was a British Asian who had always lived in Birmingham and had an in-depth knowledge of the Mirpuri community in that area. She tried different strategies in order to encourage greater participation, particularly as she recognised her cultural differences in terms of age and language:

*"My age often acts as a limitation for me especially in relation to this type of group so the aim was to reverse the roles in some sense so that the respondents didn't feel over powered by a younger female which would have potentially posed as a problem. The idea ...was to make myself out to be the typical culturally lacking third generation girl that wasn't too hot on South Asian languages (and because of my age that was understandable) so the respondents felt like they were in the driving seat and they were teaching me Mirpuri*"

The male Sylheti researcher was older and had only recently moved to the U.K. However he was also aware of the potential for difficulties in engaging with members of the opposite gender and ensured he had a female member of the Sylheti community in attendance as an 'assistant' during the female focus group sessions. As one Sylheti woman stated:

"*See... religiously we are conservative.... We do not feel comfortable with male doctors....we have a religious obligation also..... female doctors are good for us.....we can freely discuss our problems with them"*.

Telephone recruitment was found to be easier, with twice as many people being contacted this way. This negated the need to find an opportune moment during a busy clinic to talk to potential participants, and a positive rapport could be developed in confidence. As Figure 1 shows, although the total number of contacts was greater in the Mirpuri group, actual recruitment rates were lower in this group compared to those who spoke Sylheti. Reasons for declining to participate included living too far away from the hospital, going on holiday, and not able to give the time for all the focus group meetings. None of those approached voiced any objections to any of the screening questions being asked.

### Audio patient information and informed consent

Although all participants were given an audio-recording of the patient information sheet prior to the first focus group meeting, not everyone listened to it. This was particularly the case for the Mirpuri women, only one of whom was literate in both Urdu and English and reported reading the patient information sheet. The other respondents in this group (7/8) all reported relying on relatives to read the information out to them. This was in contrast to the Sylheti women, two-thirds (4/6) of whom reported listening to the audio information prior to attending the first focus group.

"*I did listen to your information audio and the sheet in Bengali as well...I found the audio one excellent...it was simple...you use very simple Sylhet terms...I did not have any problem understanding the audio...I can read Bengali but sometimes it is difficult to get the harder terms ... you know we feel comfortable in Sylheti*"

(Sylheti female participant).

All the Mirpuri and Sylheti men reported that they had listened to the audio-recorded information, and described the benefits of this method, including feelings of independence:

*"...this way you won't need to depend on your children anymore and you can do things for yourselves"*.

(Mirpuri male participant)

Those who had not listened to the audio information were given the opportunity to do so prior to the first focus group commencing. All participants gave their verbal, audio-recorded consent at this time. None of the participants had ever given audio-recorded consent before, but this method of obtaining consent was found to be acceptable to all those taking part. Two Mirpuri participants described being 'bored' when listening to the audio tape. No difficulties with understanding the audio information were reported. This was the case in both those who were literate as well as for those who were non-literate, and also for those who had previously given written consent and those who had not.

### Attrition

Although 26 Sylheti and 23 Mirpuri respondents initially agreed to participate, not all these individuals attended the focus group meetings (see Figure 1). Of those individuals who attended the focus group sessions, mean age ranged from 49 years (range 39–53) in the Mirpuri women, 52 years (range 40–71) in the Sylheti men, 54 years (range 39–71) in the Sylheti women, to 57 years (range 48–66) in the Mirpuri men. Time since diagnosis of diabetes ranged from 9 years in the Mirpuri women (range 1–16) and the Sylheti men (range 3–17), 12 years (range 4–23) in the Mirpuri men, and 14 years (range 4–28) in the Sylheti women.

Attrition was greatest in the Mirpuri women, with 50% (4/8) attending only the first two sessions before withdrawing from the study. Attempts to increase the number of people attending the focus groups included inviting associates of the original focus group members (snowballing) and this was partly successful. For the Sylheti groups, 1 woman and 1 man did not attend the first 2 focus group sessions but attended sessions 3, 4 and 5 after being fully briefed. Four further individuals (2 Mirpuri women and 2 Mirpuri men) attended the final two Mirpuri focus group sessions, however they did not fully participate in the focus group activities. Reasons for non-attendance included attending religious festivals, going abroad, ill health and difficulty arranging transport. No difficulties were reported in terms of participating in focus groups which included both literate and non-literate individuals.

A total of 9 Sylheti (5 men, 4 women) and 3 Mirpuri (all women) participants attended the end-of-project lunch, along with members of the investigative team and the diabetes service providers including the Link Workers and the Diabetes Specialist nurse. Those from the Sylheti groups were very keen to demonstrate their support for our research and suggested ways to assist in recruitment, for example through advertising in local shops and enlisting the help of shopkeepers and mosque leaders (including themselves) as 'advocates' for research participation. All those who attended the lunch were agreed that this work was an important way of encouraging greater participation in diabetes self-care and a key way of improving knowledge about diabetes. We suspect that this social and participatory aspect of the research was a key factor in their enthusiasm.

"*I just wanted to say thanks....and I am very happy to see that you are thinking about us...putting value to our Sylheti community... our culture...you know we Sylheti people feel proud if someone gives importance to our identity, our values and our culture ..."*

## Discussion

This research has highlighted some of the difficulties as well as the possibilities of conducting research with members of minority ethnic communities. In particular we found it more difficult to recruit and sustain participation in those individuals who spoke Mirpuri. Ways of maintaining interest and motivation still require developing, even when research assistants are able to engage with research participants through a common language. A previous report has also highlighted difficulties in recruiting individuals from minority ethnic communities and suggests that a range of methods may be necessary in order to maximise response rates [21]. At present however, little information is available for researchers in this regard. Our methods have the potential for use in other study populations where literacy is an issue; however they require further investigation in a larger study.

This study has its limitations, including the possible effect of small numbers of participants. Participation rates may have been influenced by previous involvement in research and therefore increased familiarity with the research process and therefore, may not represent the general population of these two ethnic groups. Other factors, for example access to transport, caring or other household responsibilities, may have also influenced participation. However, socio-demographic information such as this was not collected for all those approached in this study. It is also not known whether degree of fluency in either Sylheti or Mirpuri has had any impact on the results of this study, as no formal assessment of fluency was conducted. Greater discussion with potential participants might have avoided some of the subsequent pitfalls we experienced, for example the use of CDs rather than audio-tapes, recruitment methods, and issues which might have deterred potential participants, such as timing, venue, length of and number of sessions.

Engaging with minority ethnic communities in research, as this study has shown, can be a positive experience, but is not without its difficulties. Different perceptions of skills, knowledge or expertise (and power) may exist on the part of professional groups and lay members of the community and can be problematic [22]. Johnson [23] argues that most research involves lay people only in terms of their use as fieldworkers or research staff, employed because of the ability to speak a particular language or gain access to certain groups. Our attempts to engage with local minority ethnic communities went a step further than this, with open discussion between the research participants and all the members of the investigative team. This study incorporated time for reflection and discussion between the investigators, the two research assistants, and the service providers, with regular meetings between them all. This facilitated the sharing of information and the taking on board of alternative perspectives with regards to the research process, the content of the screening questionnaire, patient information sheets and so on. The research assistants attempted to immerse themselves in the activities of the diabetes clinic, shadowing the Link Workers, and spending time with other members of the clinic staff, so that they became accustomed to the patient experience and gained insights into diabetes care from a service-user perspective. This increased knowledge and understanding was vital in order for the research assistants to optimize their engagement with the focus group participants. Our end-of-project lunch was also evidence of the development of a partnership between researchers, service providers and service users/researched people.

This research has demonstrated the acceptability of using audio-recorded patient information sheets and obtaining informed consent via audio-recordings in minority ethnic groups where there may be difficulties with literacy, or where the main language used is spoken and does not have an agreed written form. Although our study participants had not previously encountered this method of obtaining informed consent, it was found to be acceptable by all, regardless of ability to read or write any language. However not all participants listened to the audio information at home, prior to attending the focus groups, and this was particularly the case for the Mirpuri women. It is not clear whether this was because they did not have access to an audio-cassette player, or whether their preference was to ask a relative to read out the information to them. Any future study could overcome this issue to a certain extent by using CD's rather than audio-cassettes as it is more likely that most households would have access to a CD player. One benefit of using audio-recorded information may simply be with regard to having a choice of format available to prospective participants; however this could increase participation in research by individuals who have literacy difficulties. There is evidence of a preference in South Asian populations for audio presentation of health promotion information [24]. Although there is no reason to doubt that this would carry across to informed consent issues clearly this requires confirmation in future studies.

Obtaining informed consent, and the nature of that consent, has begun to receive increased attention in particular in biomedical research but also in the social sciences [19,25]. The practice of consent-taking, as it is currently recommended, may not be universally appropriate for all populations [19]. Previous research has indicated that some minority ethnic groups favour the use of verbal as opposed to written information when participating in research [18]. This issue requires further investigation and should compare audio methods with other ways of obtaining informed consent. Following a survey of Asian language and communications [26] the Communication and Survey Policy Studies Institute recommended the use of audio media for collecting data from minority ethnic communities. However to our knowledge this form of data collection remains seldom used, and may currently be limited to small-scale or qualitative research. Indeed attitudes to participation in large clinical trials may not differ according to ethnicity, but there remain cost implications to the inclusion of non-English speaking/writing individuals in such research [27].

Our translation procedures included obtaining an academic or formal, and a lay version of the patient information. We chose to bias the final version of the information towards the lay translation as it was felt to be more easily understood and would be more acceptable to our research participants. This was indeed found to be the case and supports recent arguments for a greater focus on 'understood consent' rather than informed consent [14]. Our research also supports previous studies which have highlighted the importance of acknowledging conceptual differences in the content of information provided to research participants and utilised in research [28].

Focus groups were considered to be the most appropriate method of eliciting the views of members of the communities we were trying to reach as this did not require literacy skills. Those recruited to this study did not voice any objection to this method of research, supporting previous studies which have suggested that focus groups are an appropriate way of researching the views and experiences of minority ethnic groups [29,30]. Other methods that have been favoured include the promotion of community participation through semi-structured interviewing carried out by members of the community [31]. Individual interviews were considered less appropriate as it could place unnecessary pressure on individuals in terms of literacy skills or knowledge of diabetes. Family interviews were not seen as an appropriate alternative, due to the cost/time implications and the potential for only one or two members of the family to have Type 2 diabetes and therefore be eligible for the study. To date there has been little published health research in minority ethnic groups, in particular those whose main language is not written and where levels of literacy potentially exclude them from participating. In our study no adverse effect of including literate and non-literate individuals together in the same focus groups was observed. Furthermore there is a dearth of literature on obtaining informed consent for research purposes, except where it has been noted that non-literate individuals are excluded from participation [24]. By taking a participatory approach and working closely with both researchers *and *service users who can represent the views and opinions of the groups being researched, it is possible to develop more appropriate services and gain a greater understanding of the issues involved when attempting to promote health.

## Conclusion

This research has demonstrated that audio-recorded methods of obtaining informed consent are an acceptable alternative to written consent in study populations where literacy skills are variable. Difficulties in securing participation in research studies may be encountered, and a range of ways of engaging with minority ethnic groups should be considered. Further understanding of reasons for non-participation is required in order to improve recruitment rates. These findings have implications for widening participation in research studies, as well as in terms of service user involvement in health care more generally.

## Competing interests

The author(s) declare that they have no competing interests.

## Authors' contributions

CL was the principal investigator of the study, conceived of the study, participated in the design of the study, co-ordinated the study, participated in the analysis of the data, and drafted the manuscript. MJ was a co-investigator of the study, participated in the design of the study, the analysis of the data and assisted in the writing of the manuscript. SM was a co-investigator of the study, participated in the design of the study, the analysis of the data and assisted in the writing of the manuscript. JS was a co-investigator of the study, participated in the design of the study, the analysis of the data and assisted in the writing of the manuscript. GC was a co-investigator of the study, participated in the design of the study, the analysis of the data and assisted in the writing of the manuscript. TR was a researcher on the project, collected the data, participated in the analysis of the data and critically reviewed the manuscript. RB was a researcher on the project, collected the data, participated in the analysis of the data and critically reviewed the manuscript. AH was a co-investigator of the study, participated in the design of the study, and assisted in the writing of the manuscript. All authors read and approved the final manuscript.

## Pre-publication history

The pre-publication history for this paper can be accessed here:


